# Role of N6-methyladenosine Modification in Cardiac Remodeling

**DOI:** 10.3389/fcvm.2022.774627

**Published:** 2022-02-10

**Authors:** ManTing Choy, Ruicong Xue, Yuzhong Wu, Wendong Fan, Yugang Dong, Chen Liu

**Affiliations:** ^1^Department of Cardiology, The First Affiliated Hospital of Sun Yat-sen University, Guangzhou, China; ^2^National Health Commission (NHC) Key Laboratory of Assisted Circulation, Sun Yat-sen University, Guangzhou, China

**Keywords:** cardiac remodeling, m6A modification, lncRNAs, heart failure, epigenetic modifications

## Abstract

Cardiac remodeling is the critical process in heart failure due to many cardiovascular diseases including myocardial infarction, hypertension, cardiovascular disease and cardiomyopathy. However, treatments for heart failure focusing on cardiac remodeling show relatively limited effectiveness. In recent decades, epitranscriptomic modifications were found abundantly present throughout the progression of cardiac remodeling, and numerous types of biochemical modifications were identified. m6A modification is the methylation of the adenosine base at the nitrogen-6 position, and dysregulation of m6A modification has been implicated in a wide range of diseases. However, function of m6A modifications still remain largely unknown in cardiac diseases, especially cardiac remodeling. LncRNAs are also shown to play a vital role in the pathophysiology of cardiac remodeling and heart failure. The crosstalk between lncRNAs and m6A modification provides a novel prospective for exploring possible regulatory mechanism and therapeutic targets of cardiac remodeling. This review summarizes the role of m6A modification in cardiac remodeling in the current researches.

## Introduction

Cardiac remodeling is a physiologic and pathologic condition that may occur after various cardiovascular diseases including myocardial infarction, hypertension, cardiovascular disease and cardiomyopathy (1). Cardiac remodeling is a dynamic process in which the heart changes in size, mass, geometry and function in response to mechanical stress such as pressure or volume overload, neurohormones or cytokines in order to maintain hemodynamic homeostasis. When cardiac remodeling progresses, the transition from possible adaptive to maladaptive remodeling occurs, which may likely to establish heart failure (HF) eventually. Treatment for cardiac remodeling have been focused on neurohormonal inhibition such as renin-angiotensin-aldosterone system (RAAS) or sympathetic nervous systems (SNS) inhibition, but the proportion of poor prognosis of HF remained high. As a result, understanding the underlying pathophysiological processes involved in cardiac remodeling is critical for developing novel therapeutic strategies.

Epigenetic modifications are abundantly present throughout the physiological processes of life. It is characterized by the modification in temporal and spatial expression patterns of chromatins and genes driven by particular enzymes, without changing the nucleotide sequence of DNA and subsequent functional alterations of heritable gene (2, 3). RNA modification, also term epitranscriptomic modification, is one of these epigenetic modifications. So far, at least 170 types of RNA modifications have been discovered (4). N6-methyladenosine (m6A) is one of the abundant mRNA modifications in almost all eukaryotes. It refers to methylation of the adenosine base at the nitrogen-6 position (5). It is written by a complex of m6A methyltransferase, erased by m6A demethylase, and read by m6A binding proteins. m6A modifications govern RNA processing, including splicing, nuclear exports, RNA stability and translation, by recognizing m6A binding proteins (6). m6A has been proved to be involved in the regulation of a wide variety of pathological processes including obesity, metabolic disease and carcinogenesis. For example, one of the m6A component, the RNA methyltransferase METTL3, was discovered to promote proliferation and invasion of human lung cancer cells (7). As for cardiovascular disorders, continuous dynamic control of m6A have been shown to play a critical role as well. METTL14, another methyltransferase of RNA, enhanced the m6A modification of pri-miR-19a and promoted mature miR-19a processing to increasing atherosclerosis vascular endothelial cells proliferation and invasion (8). At the moment, researches of m6A modification in the field of heart failure and cardiac remodeling are still in their initial phases. Zhang et al. revealed the different expression profiles of m6A regulators in heart failure with preserved ejection fraction (HFpEF) patients in combination with a clinical case-control study and animal experiment, and highlighted the relationship between m6A modification and the risks of HFpEF (9). Given the comprehensive regulation of m6A modification in diseases, much remains to be explored in cardiac remodeling and heart failure.

Long non-coding RNAs (lncRNAs) are non-protein coding transcripts with a length more than 200 nucleotides. LncRNAs could govern the cellular processes, such as cell cycles regulation, differentiation through diverse mechanisms like transcription, translation, splicing, etc, in various disease states (10). Emerging data have shown that lncRNAs play a key role in the pathogenesis of cardiac remodeling and heart failure (11). Many mature lncRNAs are modified after transcription, and numerous types of biochemical modifications were found in lncRNAs (12). The relationship between lncRNAs and m6A modifications still remain largely unknown, provides a fresh viewpoint for exploring the possible regulatory mechanism and suggests that m6A modification and lncRNAs interplay might be significant treatment targets for various diseases (13). This review provides an overview of recent advances in m6A modifications and gives an updated outline of the association between m6A modification and cardiac remodeling, and provide an insight into potential molecular biomarkers associated with the m6A modification of lncRNAs and therapeutic targets in cardiac remodeling.

## Machinery and Biological Roles of m6A Modification

**Figure 1:**
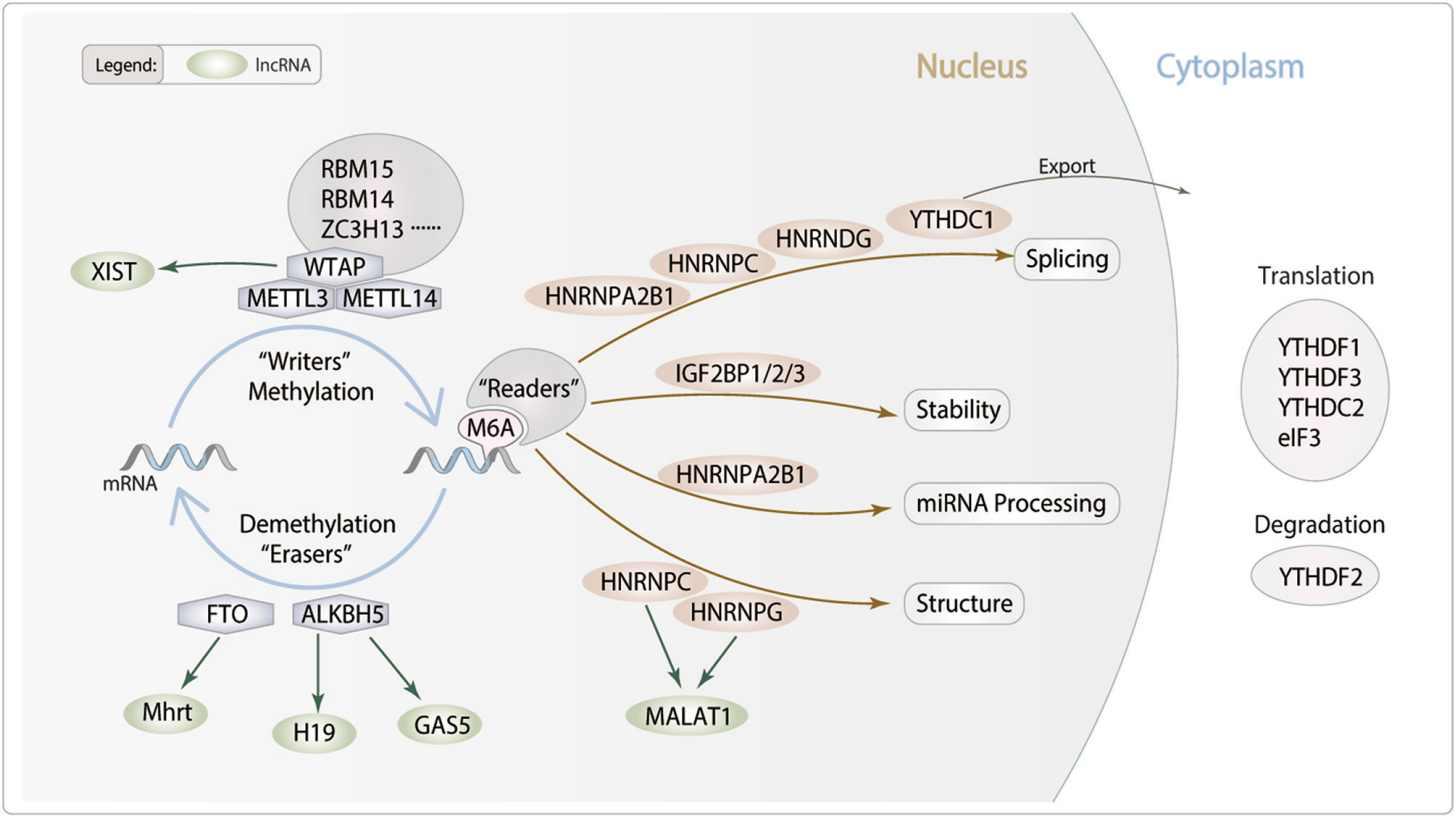
Potential role of lncRNA m6A modification in cardiac remodeling. m6A is deposited by “Writers” (METTL3/14, WTAP, RBM14/15 and ZC3H13), removed by “Erasers” (FTO and ALKBH5), and recognized by “Readers” (YTHDC1/2, YTHDF1/2/3, IGF2BP1/2/3, HNRNPA2B1, HNRNPC, HNRNPG and eIF3). m6A modifications can regulate RNA processing, including splicing, nuclear exports, stability and translation. We hypothesis lncRNA m6A modification which may modulate particular pathophysiological process of cardiac remodeling.

m6A modification is found throughout species in evolutionary patterns, having a consensus sequence of the m6A center site. Their critical roles in epigenetics and physiological connections to numerous human diseases have given them a huge scientific and medical attention. m6A modification is a dynamic and reversible posttranscriptional modification process which was implemented in three distinct kinds of protein complexes (“writer” and “reader” and “eraser” proteins) and can alter important biological processes by adding, removing, or preferring identify the m6A sites (Figure 1).

### Writer

Methyltransferase (m6A “writers”) are protein components that catalyze m6A methylation of RNAs. m6A modification is achieved by a large methyltransferase complex (MTC). The m6A MTC mainly consists of the methyltransferase-like proteins, such as METTL3 and METTL14, and various protein factors indispensable for their proper catalysis, including WTAP, RBM15, ZC3H13, and RBM14. METTL3 is responsible for the installation of m6A on RNA. According to a phylogenetic study, METTL14 is a homolog of METTL3, with 43% similarity. The METTL3/METTL14 form a stable heterodimer core complex exhibits far more m6A methyltransferase activity than either component alone. The METTL3/METTL14 complex preferentially methylates RNA on sites of RRACH (R = A/G, H = A/U/C) *in vitro*, in accordance with the RRACH motif distribution of m6A transcriptome-wide (14). WTAP, as the scaffold protein of m6A-MTC, exhibits an influence on the METTL3/METTL14 compound structure and substrate composition (15). Additionally, several proteins have been shown to involved in substrate binding, catalytic efficiency, stability, and localization in addition to the core of m6A complex. RBM15, ZC3H13, HAKAI, and VIRMA, for example, have been identified as WTAP components, and this association offers a scaffold for METTL3/METTL14 recruitment for methylation (16).

### Eraser

Demethylases (m6A “erasers”) are enzymes that remove the methyl group from RNA. m6A is one of the few intrinsic RNA modifications that may be reversed. The identification of RNA demethylase FTO, was the crucial breakthrough that revived the m6A function. FTO catalyzes oxidative demethylation of m6A in an Fe(II)- and α-KG-dependent manner which preferentially bind to pre-mRNA strands in intronic regions, in the proximity of alternatively spliced exons and poly(A) sites (17). FTO is a member of the ALKB family. Another family member, ALKBH5, has also been discovered as a demethylase. Both ALKBH5 and FTO are confined to nucleus and located by nuclear speckles, which show the splicing effects of methylation (18). Instead of the FTO oxidative demethylation, ALKBH5 catalytic reaction directly removes the methyl group from the m6A methylated adenosine (19). ALKBH5 found as m6A mRNA demethylase employing Fe^2+^ and 2OG cofactor, which work together to remove the methyl group in m6A containing substrates.

### Reader

The biological function of m6A modification is regulated by m6A-binding proteins, also known as the “readers,” including YTHDF1/2/3, YTHDC1/2, IGF2BP1/2/3, HNRNPA2B1 and eIF3. Reader proteins that act as functional mediators, selectively identity target m6A-modified mRNA and regulate varies of RNA metabolism processes such as RNA splicing, transport, translocation, translation and degradation (20). YTHDC1 is a nuclear protein involved in gene splicing. However, YTHDF1-3 are cytoplasmic m6A readers. YTHDF1 and YTHDF3 collaborate to influence the translation of m6A-containing mRNAs, whereas YTHDF2 speeds up mRNA decay and YTHDC1 influences the nuclear processing of its targets (21). Moreover, a number of recent papers claim to have discovered the presence of additional sorts of readers. IGF2BPs (IGF2BP1-3), HNRNPA2B1 and eIF3 can affect the splicing, translation, stability and degradation of mRNA (21). IGF2BPs belong to a conserved family of RNA-binding, and associate with target mRNAs in cytoplasmic ribonucleoprotein complexes, and enhance the stability and storage of their target mRNAs, therefore regulating the output of gene expression (22). HNRNPA2B1 can directly binds nuclear transcripts to induces alternative splicing effects and boosting microRNA processing (23). eIF3 plays an important role in recruitment of the pre-initiation complex PIC to mRNA. Moreover, YTHDF1 relocates from the cytoplasm to the nucleus and regulates translation may depends on interaction with eIF3 (24).

## m6A Methylation in Cardiac Remodeling

Cardiac remodeling involves almost all the cell types in the heart including cardiomyocytes, cardiac fibroblasts, vascular smooth muscle cells and vascular endothelial cells. Remodeling encompasses cellular changes including cardiomyocytes hypertrophy, necrosis, apoptosis, vascular differentiation and fibroblast proliferation. Recently, it was discovered that m6A methylation plays an important role in mediating significant structural alterations in the failing heart. Researchers observed that the overall level of m6A modification of the transcripts in the healthy mouse and human heart is increased by using next-generation sequencing. Moreover, the changes in m6A methylation exceeded changes in gene expression with the course of heart failure in both mice and humans (25).

### Cardiomyocytes Hypertrophy

Cardiac hypertrophy is one of the predominant components of cardiac remodeling. During the early stages of stress, cardiac hypertrophy serves as a compensatory mechanism occurred in cardiomyocytes, manifested as myocytes hypertrophy, and progressive thickening of the ventricular wall. Sustained pathological hypertrophy, on the other hand, is a major cause of cardiomyocytes remodeling and HF. In addition to the transcriptional control of gene expression during hypertrophy, posttranscriptional regulation of protein expression is increasingly recognized as a vital mechanism for hypertrophic control (26) and m6A methyltransferase METTL3 is one of them (Table 1). Investigation showed that the level of m6A modification was significantly increased in isolated neonatal rat ventricular cardiomyocytes responded to hypertrophic stimulation by using m6A immunoprecipitation followed by RNA sequencing (27). In order to further investigate the possibility that m6A is involved in the regulation of cardiomyocyte homeostasis and hypertrophy *in vivo*, METTL3-overexpressing mice were studied and showed significant cardiac hypertrophy when exposed to pressure overload stress. However, results did not show accelerated cardiac dysfunction. METTL3 knockout mice, on the other hand, revealed indications of failure on both morphological and functional levels (27). These evidences implied that the METTL3-m6A pathway could be a novel critical regulator of cardiac homeostasis. Interestingly, opposite findings were revealed by Kmietczyk et al. (28). In compared with healthy myocardium, their investigation indicated a higher METTL3 activity and a predominance of RNA transcripts enriched in m6A in human failing heart and dilated cardiomyopathy. Additionally, overexpression and knockdown of METTL3 expression affected the cellular size and cardiomyocytes remodeling both *in vitro* and *in vivo*. Mechanically, according to Dorn et al. (27), m6A peaks were selectively abundant in mRNAs encoding protein kinases and modifiers, such as mitogen-activated protein kinases (MAPKs) family, resulting in the significant increase of cardiomyocyte size. On the other side, Kmietczyk et al. (28) suggested the mRNA methylation is highly dynamic when exposed to stress conditions, which in turn to regulate translational efficiency by modifying transcript stability. These two findings appear to be contradictory, and previous researches have revealed that the genetic background of mice may influence the phenotypic outcome of cardiac disorders post-intervention. It is possible that variances in methodologies and mice strain backgrounds which may explain some of the disparities in the data. However, this also highlights that the underlying mechanisms are highly uncertain, and further investigations would be necessary.

**Table 1:** Role of m6A modification in cardiac remodeling.

**Cardiac remodeling**	**m6A regulators**	**m6A levels**	**Cell (tissue) types**	**Main functions and mechanisms**	**References**
Cardiomyocytes hypertrophy	METTL3 upregulated	Increased	Cardiomyocytes under hypertrophic conditions.	METTL3 promotes the expression of MAPKs family in cardiomyocytes.	(27)
	METTL3 upregulated	Increased	Human failing myocardium.	m6A regulates translational efficiency by affecting transcript stability.	(28)
	FTO upregulated	Decreased	Cardiomyocytes by leptin stimulation.	FTO upregulation *via* JAK2/STAT3-dependent CUX1 upregulation.	(29)
	FTO downregulated	Increased	Cardiomyocytes.	FTO regulates Intracellular Ca2^+^ and sarcomere dynamics in cardiomyocytes.	(34)
Cardiomyocytes death	METTL3 upregulated ALKBH5 downregulated	Increased	H/R-treated cardiomyocytes and I/R-treated mice heart.	METTL3 enhances the binding of TFEB to HNRNPD, which decreases TFEB expression, thereby impairing autophagic flux and enhancing apoptosis. ALKBH5 exerts the opposite effects.	(32)
	FTO downregulated	Increased	Failing human (both ischemic and non-ischemic), post-MI pig and mouse hearts.	FTO regulated SERCA2A, MYH6/7 and RYR2 expression and prevented cardiac contractile transcription deterioration.	(34)
Extracellular matrix remodeling	METTL3 upregulated	Increased	Cardiac fibroblasts treated with TGF-β1 and in the chronic MI murine hearts.	METTL3 promotes cardiac fibrosis through Smad-mediated pathway.	(35)
	METTL3 upregulated	Increased	Mice hearts.	METTL3 overexpression following TAC operation decreases fibrosis and collagen transcription.	(28)
	FTO upregulated	Decreased	Murine MI hearts.	Reduces scar size.	(34)
Vascular remodeling	METTL3 upregulated	Increased	ADSCs undergoing VSMCs differentiation induction.	Stimulates the differentiation of ADSCs into vascular VSMCs and regulates the secretion of VEGF, HGF, TGF-β, GM-CSF, bFGF, and SDF-1.	(38)
	FTO upregulated	Decreased	Murine MI hearts.	Reduces cardiac fibrosis (decreases of scar size%) and increases angiogenesis (higher number of CD31− positive cells).	(34)
	METTL14 upregulated	Increased	TNF-α stimulated HUVECs.	METTL14 modifies FOXO1 mRNA to promote TNF-α-induced endothelial monocyte adhesion.	(39)
	METTL14 upregulated	Increased	ASVEC.	METTL14 regulates the maturation of pri-miR-19a, to promotes invasion and proliferation of cardiovascular ECs.	(8)

*m6A, N^6^-methyladenosine; METTL3, methyltransferase-like 3; FTO, fat mass- and obesity-related protein; MAPKs, mitogen-activated protein kinases; JAK2, Janus kinase 2; STAT3, signal transduction and activator of transcription 3; CUX1, Cut Like Homeobox 1; MI, myocardial infarction; TFEB, Transcription Factor EB; H/R, hypoxia/reoxygenation; I/R, ischemia/reperfusion; ALKBH5, AlkB Homolog 5; HNRNPD, Heterogeneous Nuclear Ribonucleoprotein D; SERCA2a, sarcoplasmic/endoplasmic reticulum Ca^2+^-ATPase 2a; MYH6/7, β-myosin heavy chain 6/7; Ryr2, ryanodine receptor 2; TAC, transverse aortic constriction; ADSCs, adipose-derived stem cells; VSMCs, vascular smooth muscle cells; VEGF, vascular endothelial growth factor; HGF, hepatocyte growth factor; TGF-β, transforming growth factor β; GM-CSF, granulocyte-macrophage colony-stimulating factor; bFGF, basic fibroblast growth factor; SDF-1, stromal cell-derived factor-1; METTL14, Methyltransferase like-14; HUVECs, Human umbilical vein endothelial cells; ASVEC, atherosclerotic vascular endothelial cells; FOXO1, forkhead box O1; ECs, endothelial cells*.

As for FTO, a well-known m6A demethylase protein, has been linked to cardiac hypertrophy and muscular contraction in cardiac remodeling (Table 1). Gan et al. revealed that nuclear FTO expression in cardiomyocytes was considerably increased during an adipokine induced cardiomyocyte hypertrophic response in newborn rat cardiomyocytes, leptin-induced FTO upregulation in cardiomyocytes *via* JAK2/STAT3-dependent CUX1 upregulation (29). The researchers also discovered that FTO knockdown reduced the hypertrophic response *in vitro*, indicating that FTO plays an essential regulatory function in cardiac hypertrophy.

### Cardiomyocytes Death

Recently, the role of apoptosis in the heart during ischemic and non-ischemic cardiomyopathies has been explored to involved in cardiac remodeling and heart failure. Structural or functional alterations in the myocardium may be intimately associated with myocardial ischemia. Alterations in blood supply that occur over time may result in changes in cardiac tissue which may have impacts on both short- and long-term cardiovascular function and prognosis. Myocardial infarction causes widespread cardiac damage *via* ischemic and ischemia/reperfusion injury, resulting in a deleterious effect on cardiomyocytes and cardiac functions. Cardiomyocyte apoptosis and necrosis, is a hallmark characteristic features of myocardial infarction and cardiac remodeling. Autophagy is triggered during ischemic stress to protect cardiomyocytes from ischemic or ischemia/reperfusion injury. However, excessive autophagy activation may have a deleterious effect on the heart in reperfusion and other stress circumstances, revealing the controversial nature of autophagy in myocardial ischemia. In numerous animal models, blocking apoptosis or regulating autophagy process could effectively reduce cardiac remodeling and heart failure (30, 31).

Song et al. (32) identified that in hypoxia/reoxygenation (H/R)-treated cardiomyocytes as well as the ischemia/reperfusion (I/R)-treated mice heart, the m6A methylations were increased owing to the upregulation of METTL3. Silencing METTL3 enhanced autophagic flux and inhibited apoptosis in H/R-treated cardiomyocytes. Whereas the RNA demethylase ALKBH5 acted in a opposite manner during myocardial I/R (32). Mechanistically, METTL3 methylates the transcription factor EB (TFEB), a critical regulator of lysosomal biogenesis and also stimulated the expression of autophagy genes (33). Silencing of METTL3 promoted TFEB expression and its nuclear translocation. Furthermore, TFEB can reduce METTL3 expression by decreasing the stability of the METTL3 mRNA. TFEB knockdown eliminated the enhanced autophagy driven by METTL3 downregulation, demonstrating that METTL3 mediates autophagic flux in a TFEB-dependent manner (32). Although authors did not evaluate changes to the overall m6A levels following ALKBH5 dysregulated, they demonstrated a vital role for m6A in autophagy regulation in H/R cardiomyocytes (Table 1).

Besides, Mathiyalagan et al. (34) demonstrated the m6A modifications were increased in failing human (both ischemic and non-ischemic), post-MI pig and mouse hearts and hypoxic cardiomyocytes, resulting in a decrease in contractile performance. To identify the m6A regulators in the ischemic myocardium, the expressions of the major methylases, demethylases, and reader proteins were measured. They found FTO expression was significantly decreased in failing mammalian hearts and hypoxic cardiomyocytes. It is remarkable that loss of FTO exhibited a significantly increased number of arrhythmic events which are mediated primarily by cardiac contractile genes regulation, such as SERCA2A, MYH6/7 and RYR2, resulting in contractile dysfunction in cardiomyocytes. *In vivo*, FTO overexpression improved cardiac function remarkably during the chronic stage of post-myocardial infarction (34). FTO prevented cardiac contractile transcription deterioration and enhanced protein expression by selective demethylation under ischemic circumstances, thereby safeguarded cardiomyocyte contractile function by regulating intracellular Ca^2+^ dynamics (34).

### Extracellular Matrix Remodeling

Cardiac fibrosis, defined by pathological activation of cardiac fibroblasts and excessive buildup of extracellular matrix (ECM) in the afflicted tissue. Cardiac fibroblasts are activated and transform into myofibroblasts which produce a large amount of matrix metalloproteinases (MMPs), leading to accumulation and composition alteration of ECM. In the cardiac remodeling, cardiac fibrosis contributes to the dysfunction of failing hearts by making the heart stiffer and reducing its function due to its inability to contract or conduct electric impulses required for the transmission of the contraction. The activation and differentiation of cardiac fibroblasts are also influenced by m6A methylation. Increased METTL3 expression was shown in cardiac fibroblasts treated with TGF-β1 and in the murine hearts suffered from chronic myocardial infarction (35). METTL3 overexpression promoted cardiac fibrosis, fibroblast-to-myofibroblast transition, and enhanced extracellular matrix production and accumulation, whereas METTL3 silencing had the opposite effect. *In vivo*, silencing METLL3 significantly suppressed cardiac fibrosis progression. Smad2/3 are key regulators of the fibrogenesis process in cardiac fibroblasts, whereas silencing METTL3 reduced the upregulation of Smad2/3 induced by TGF-β1, suggesting that METTL3 regulated cardiac fibrosis at least partially through Smad-mediated pathway (35). Consistent with these results, Kmietczyk et al. (28) carefully analyzed and revealed that AAV9-mediated METTL3 overexpression following TAC operation significantly decreased fibrosis and collagen transcription in mice hearts. In addition to modifying cardiomyocyte hypertrophy, AAV9-mediated METTL3 overexpression may partially modulate the cardiac fibroblast phenotype. These evidences indicated METTL3-mediated m6A methylation played a critical role in cardiac fibrosis regulation (Table 1).

In addition, along with the positively regulating contractile protein expression, FTO-dependent demethylation was also indicated to affected critical non-contractile processes involving tissue morphogenesis, angiogenesis, extracellular matrix organization, fibrosis, and cell proliferation and differentiation in murine MI hearts (34). In FTO-overexpressing mice post-MI models, fibrosis in mice overexpressing FTO was significantly decreased compared with their respective controls, as measured by scar size (%) (34).

### Vascular Remodeling

Vascular remodeling refers to alterations in the structure of resistance vessels contributing to elevated systemic vascular resistance. It is a dynamic structural change that involves alterations to numerous cellular including endothelial cells (ECs), vascular smooth muscle cells (VSMCs), and fibroblasts and non-cellular components, which may lead to vascular stiffness. During vascular remodeling and heart failure, abnormalities in ECs and VSMCs proliferation and migration, endothelial dysfunction, inflammatory processes, and the production or breakdown of extracellular matrix components are present and predominant (36, 37). METTL3 and FTO have been shown to be involved in the regulation of vascular or cardiac dysfunction under stress conditions (Table 1). Under hypoxia stimulations, METTL3 can stimulate the differentiation of adipose-derived stem cells into vascular VSMCs (38). Also, evidences showed that overexpression of FTO in the ischemic myocardium could reduce cardiac fibrosis and increase angiogenesis (34). For angiogenesis, anti-CD31 immunostaining was performed by Mathiyalagan et al. (34). They measured the angiogenic response by detecting CD31-positive endothelial cells in the infarct border zone of murine hearts 4 weeks after MI, and discovered FTO-overexpressing hearts had a much higher proportion of CD31-positive cells than their respective control group (34). METTL14, as a methylase, was discovered to stimulate inflammatory response in ECs and promote atherosclerotic plaque formation by interacting with forkhead box O1 (FOXO1). Through YTHDF1 recognition, METTL14 enhanced the translation of FOXO1 mRNA, therefore increased the expression of the endothelial adhesion molecules VCAM-1 and ICAM-1, which promoting endothelial adhesion (39). In addition, researchers revealed that METTL14 mediated the m6A modification of pri-miR-19a, promoted its processing and maturation, which promoting the invasion and proliferation of cardiovascular endothelial cells (8).

### LncRNAs in Cardiac Remodeling

Numerous investigations have revealed that long non-coding RNAs played critical regulatory roles under stressful situation: lncRNAs Mhrt, XIST, MIAT, Chast, CHRF, ROR, H19, and Plscr4 are involved in myocardial hypertrophy, whereas XIST, MALAT1, GAS5, Neat1, AK139328, APF, CAIF, AK088388, CARL, HOTAIR, and NRF are involved in cardiomyocytes apoptosis and necrosis. Moreover, MIAT, MALAT1, Wisper, MEG3, and H19 are involved in extracellular matrix reconstruction. In addition, MALAT1, GAS5, H19, TUG1, AK098656, TRPV1, Giver, and Lnc-Ang362 have been implicated in vascular remodeling (11, 40). m6A modification has been identified in long non-coding RNAs by methylated RNA immunoprecipitation sequencing (MeRIP-seq) m6A on lncRNAs accounts for ~12% of the total m6A peaks (41). The m6A methylation of lncRNAs has been demonstrated to modify their structure (42), stability (43), transport and destruction (44), hence altering the biological processes or the interaction with other RNA molecules (45–47). However, only a few of m6A modification on lncRNA have been reported in cardiac diseases. In this section, we will focus on the potential function of m6A of lncRNAs in cardiac remodeling (Table 2).

**Table 2:** Potential role of lncRNA m6A modification in cardiac remodeling.

**LncRNAs**	**LncRNAs in cardiac remodeling**	**m6A regulators**	**LncRNAs modified with m6A**
Mhrt	Mhrt regulated the expression of KLF4 to prevent ERK and KLF4 interaction, hence inhibiting the development of cardiac hypertrophy (48).	FTO	FTO downregulated in heart failure mouse model. FTO overexpression increased the expression of Mhrt, which inhibited the apoptosis of cardiomyocytes induced by the H/R (50).
	Mhrt protects cardiomyocytes from apoptosis against the H_2_O_2_ or H/R exposure (49).		
XIST	XIST could modulate the progression of cardiomyocyte hypertrophy by regulating miR-330-3p/S100B pathway and miR-101/TLR2 axis (53, 54).	RBM15/ RBM15B, WTAP, METTL3	XIST is highly methylated with at least 78 m6A residues (45). m6A methylation of XIST creates a multiprotein complex and recruits the silencing complex, thereby promotes XIST-mediated transcriptional repression (45).
	Overexpression of XIST promoted cardiomyocyte apoptosis and inhibit proliferation by mediating PDE4D expression *via* targeting miR-130a-3p (55).		
	In acute myocardial infarction, XIST regulated expression of anti-apoptotic biomarkers Bax, hexokianse 2 and Notch1 (56, 57).		
MALAT1	MALAT1 could increase cardiomyocytes proliferation or apoptosis in myocardial I/R rats through activating PI3K/Akt and β-catenin signaling pathways (59, 60).	/	SCARLET verified MALAT1 was also highly modified by m6A and contained several m6A motifs (A2515, A2577, A2611, and A2720) (66).
	MALAT1 promoted cardiomyocyte apoptosis of HL-1 or H9c2 cells under H/R conditions *via* interacting with microRNAs (61, 62).	HNRNPC and HNRNPG	The binding of MALAT1 and the m6A “reader” HNRNPC and HNRNPG would increase if A2577 and A2515 of MALAT1 modified by m6A, thereby alter the expression of MALAT1(67).
	MALAT1 promoted human endothelial cells pyroptosis by affecting NLRP3 expression through competitively binding miR-22 (63). MALAT1 mediated cardiac fibrosis in MI mice model (64).		
GAS5	GAS5 hasten myocardial I/R injury by sponging miR-532-5p in myocardial cells (68).	ALKBH5, YTHDF2	ALKBH5 inhibited m6A modification of GAS5 to its stability. Also, m6A promoted the degradation of GAS5 in a YTHDF2-dependent manner (70).
	GAS5 knockdown would aggravate microvascular dysfunction by altering β-catenin signaling activity (69).	YTHDF3	Silencing m6A “reader” YTHDF3 enhances the degradation of GAS5 (44).
H19	H19 promoted myocardial apoptosis (71). H19 overexpression promoted VSMCs proliferation and inhibited its apoptosis (72).	ALKBH5	ALKBH5 regulated the expression of H19 by mediating its m6A modification levels in H9c2 cells with H_2_O_2_-induced senescence (73).

LncRNA myosin heavy chain associated RNA transcript (Mhrt) have been proven in regulating the cardiac remodeling. Mhrt is related to heart failure due to its ability to modulate cardiac hypertrophy. Mhrt increased the expression of KLF4 through direct binding to miR-145a-5p or inhibiting phosphorylation of KLF4, to prevent ERK and KLF4 interaction, hence inhibiting myocardin expression and the development of cardiac hypertrophy (48). Besides, Mhrt could protect cardiomyocytes from apoptosis against the H_2_O_2_ or H/R exposure (49). Shen et al. (50) observed that the expression of FTO and Mhrt were downregulated in heart failure mouse model. FTO overexpression inhibited the m6A modification of Mhrt, hence increased the expression of Mhrt, which inhibited the apoptosis of cardiomyocytes induced by the H/R. These results indicated that m6A modification of Mhrt participates in the development of cardiac disease (50).

LncRNA X-inactive specific transcript (XIST), plays a critical role in the regulation throughout the whole spectrum of human diseases and can be utilized as a novel diagnostic and prognostic biomarker for human disease (51, 52). XIST was investigated positively regulates S100B expression and enhance TLR2 expression, thereby modulates the progression of cardiomyocyte hypertrophy by miR-330-3p/S100B pathway and miR-101/TLR2 axis (53, 54). XIST was up-regulated in cardiomyocytes after infarction. Overexpression of XIST might promote cardiomyocyte apoptosis and inhibit proliferation by mediating PDE4D expression *via* targeting miR-130a-3p (55). In acute myocardial infarction, XIST protected hypoxia-induced cardiomyocyte injury and repressed myocardial apoptosis by interacting directly with various miRNAs and positively regulated expression of anti-apoptotic biomarkers such as Bax, hexokianse 2, and Notch1 (56, 57). Researchers have shown XIST is highly methylated with at least 78 m6A residues. m6A methylation of XIST creates a multiprotein complex with RBM15/RBM15B, WTAP, and METTL3, which in turn recruits the silencing complex, thereby promotes XIST-mediated transcriptional repression (45).

LncRNA metastasis-associated lung adenocarcinoma transcript 1 (MALAT1) is widely expressed and highly conserved in human tissues (58). MALAT1 has been reported to increase cardiomyocytes proliferation or apoptosis in rats with myocardial I/R induced injury through activating canonical signaling pathways, such as PI3K/Akt and β-catenin signaling pathways (59, 60). Also, MALAT1 was discovered to promote cardiomyocyte apoptosis of HL-1 or H9C2 cells under H/R conditions *via* interacting with microRNAs (61, 62). LncRNA MALAT1 profoundly induced by stimuli such as hypoxia, cytokine and oxidative stress in ECs, and it could regulate various pathophysiological processes of cardiac remodeling regarding to vascular remodeling (63, 64). For example, ox-LDL is thought to be a critical factor in the initiation and progression of endothelial dysfunction. MALAT1 induced by ox-LDL was also found to protect against endothelial injury by sponging the miR-22-3p *via* activation of AKT pathway (65). Recently, several m6A-deposition sites of MALAT1 have been identified, by the method which can accurately determines m6A status at any site in mRNA/lncRNA, named SCARLET. It has been verified that lncRNA MALAT1 was also highly modified by m6A and contained several m6A motifs (A2515, A2577, A2611, and A2720) (66). m6A modifications on site A2577 and A2515 of MALAT1 destabilize the RNA hairpin of MALAT1, releasing the poly-U tract and increasing the binding with HNRNPC and HNRNPG (42, 67), indicating MALAT1 could be m6A modified to regulate the development of various diseases including cardiac remodeling.

LncRNA GAS5 was found to promote myocardial I/R injury by sponging miR-532-5p in myocardial cells (68). LncRNA GAS5 was also confirmed to participate in the vascular remodeling. Wang et al. showed that lncRNA GAS5 regulated ECs and VSMCs function, GAS5 knockdown would aggravate microvascular dysfunction by altering β-catenin signaling activity, thus increased neovascularization and capillary leakage (69). The m6A modifications of GAS5 have been found. ALKBH5 inhibited m6A modification of GAS5 to its stability. Besides this, m6A promoted the degradation of GAS5 in a YTHDF2-dependent manner (70). Additionally, it was discovered that silencing m6A “reader” YTHDF3 enhances the degradation of GAS5 (44). These findings suggests that m6A-modified GAS5 may play a role in the development of cardiac remodeling.

In recent decades, lncRNA H19, a potential serum marker for coronary heart disease, is involved in the regulation of vascular remodeling. For example, in I/R condition, lncRNA H19 is reported to largely participated and promoted myocardial apoptosis (71). Researchers have investigated that H19 overexpression promoted VSMCs proliferation and inhibited its apoptosis (72). The m6A modified-H19 has been revealed involving in the development of cardiac diseases. In H_2_O_2_-induced senescence, lncRNA H19 expression decreased and m6A modification increased following H/R, ALKBH5 regulated the expression of H19 by mediating its m6A modification levels (73).

## Conclusions and Future Prospects

In cardiac remodeling which is the fundamental step for the progression of heart failure, m6A modification shows great potential in mechanistic studies and therapeutic target explorations. However, m6A has complicated impacts on gene expression and is hard to be isolated at a cellular level. It is hard to totally remove the complex of m6A methyltransferase, as knockout typically results in cell death. Studies were significantly impeded by this situation. It will require more studies to completely illustrate how m6A impacts the *in vivo* behavior of target RNAs and its direct downstream effects on gene expression in the setting of cardiac remodeling.

It is worth noting that, the interplay of m6A and lncRNAs is prevalent and inspiring, which significantly broadening the scope of epitranscriptomic regulation. m6A modifying lncRNAs still needs to interact with other components, such as other proteins, miRNAs and mRNAs. m6A modification on lncRNAs are emerging regulators in diverse process but remained in the level of phenotype researches rather than further mechanism manifestations. In addition, it remains unknown how m6A modifications on lncRNAs differs from the those on mRNAs in cardiac remodeling. As a whole, improvements in RNA modification sequencing and mapping technologies will definitely lead to a deeper understanding of RNA epigenetics mechanisms, and encourages the development of novel molecular treatments for cardiac remodeling in the future.

## Author Contributions

MC and RX wrote the manuscript. YW drew the figure. WF, YD, and CL edited the manuscript. All authors contributed to the article and approved the submitted version.

## Funding

The present study was funded by the National Natural Science Foundation of China (No. 81770394).

## Conflict of Interest

The authors declare that the research was conducted in the absence of any commercial or financial relationships that could be construed as a potential conflict of interest.

## Publisher's Note

All claims expressed in this article are solely those of the authors and do not necessarily represent those of their affiliated organizations, or those of the publisher, the editors and the reviewers. Any product that may be evaluated in this article, or claim that may be made by its manufacturer, is not guaranteed or endorsed by the publisher.

## Abbreviations

m6A: N6-methyladenosine
LncRNAs: long non-coding RNAs
HF: heart failure
RAAS: renin-angiotensin-aldosterone system
SNS: sympathetic nervous systems
HFpEF: heart failure with preserved ejection fraction
METTL3: Methyltransferase like-3
METTL14: Methyltransferase like-14
WTAP: Wilms’ tumor 1–associated protein
RBM15: RNA Binding Motif Protein 15
ZC3H13: Zinc Finger CCCH-Type Containing 13
RBM14: RNA Binding Motif Protein 14
FTO: fat mass and obesity-associated gene
ALKBH5: AlkB Homolog 5
YTHDF/YTHDC: YT521-B homology (YTH) domain family of proteins
IGF2BPs: insulin-like growth factor 2 mRNA-binding protein
HNRNPA2B1: heterogeneous nuclear ribonucleoproteins A2/B1
eIF3: eukaryotic initiation factor 3
ECM: extracellular matrix
ECs: endothelial cells
VSMCs: vascular smooth muscle cells
ADSCs: adipose-derived stem cells
H/R: hypoxia/reoxygenation
I/R: ischemia/reperfusion
Mhrt: myosin heavy chain associated RNA transcript
XIST: X-inactive specific transcript
MALAT1: metastasis-associated lung adenocarcinoma transcript 1
MAPKs: mitogen-activated protein kinases.
